# Root length and alveolar bone level of impacted canines and adjacent teeth after orthodontic traction: a long-term evaluation

**DOI:** 10.1590/1678-77572016-0133

**Published:** 2017

**Authors:** Aldir Cordeiro da SILVA, Anderson CAPISTRANO, Renata Rodrigues de ALMEIDA-PEDRIN, Maurício de Almeida CARDOSO, Ana Cláudia de Castro Ferreira CONTI, Leopoldino CAPELOZZA

**Affiliations:** 1 Universidade do Sagrado Coração, Departamento de Ortodontia, Bauru, Brasil.

**Keywords:** Impacted tooth, Root resorption, Corrective orthodontics, Cone-beam computed tomography

## Abstract

**Objective:**

The aim of this retrospective study was to evaluate the long-term effects of orthodontic traction on root length and alveolar bone level in impacted canines and adjacent teeth.

**Material and Methods:**

Sample consisted of 16 patients (nine males and seven females), mean initial age 11 years and 8 months presenting with unilaterally maxillary impacted canines, palatally displaced, treated with the same surgical and orthodontic approach. Teeth from the impacted-canine side were assigned as Group I (GI), and contralateral teeth as control, Group II (GII). The mean age of patients at the end of orthodontic treatment was 14 years and 2 months and the mean post-treatment time was 5 years and 11 months. Both contralateral erupted maxillary canines and adjacent teeth served as control. Root length and alveolar bone level (buccal and palatal) were evaluated on cone-beam computed tomography (CBCT) images. The comparison of root length and alveolar bone level changes between groups were assessed by applying paired t-test, at a significance level of 5% (p<0.05).

**Results:**

There were no statistically significant differences in root length and buccal and palatal bone levels of canines and adjacent teeth among groups.

**Conclusions:**

Impacted canine treatment by closed-eruption technique associated with canine crown perforation, has a minimal effect on root length and buccal and palatal alveolar bone level in both canine and adjacent teeth, demonstrating that this treatment protocol has a good long-term prognosis.

## Introduction

Dental abnormalities are often found during the diagnosis of orthodontic patients, especially the ectopic eruptions^30^. Several studies have associated the canine impaction with other anomalies^1,18,27,29^, such as agenesis, microdontia and dental transpositions, pointing to the hypothesis that these events have the same genetic origin^1^. Disregarding the third molars, maxillary canines present the greatest prevalence of ectopic eruption, ranging from 1% to 3% depending on the studied population group^5-7,13^ and, specifically, the palatal displacement is more frequent than the buccal one^2^.

It is important to highlight that failure in early diagnosing and treating the impacted tooth can result in serious damages, such as external resorption of adjacent teeth esthetic problems, reduced dental arches, and increased follicular cyst formation, that may eventually cause tooth loss and periodontal involvement^9,12^.

The main side effect of orthodontic traction when managing ectopic canines is root resorption, which can affect not only canines but also adjacent teeth^20,25^. In a study using periapical radiographs to evaluate patients presenting palatally displaced canines treated by means of open surgical exposure and leveling approach, the roots of impacted canines and lateral incisors were smaller than those of contralateral teeth used as control^28^.

Factors, such as the initial positioning of the teeth, the size of the follicle and the proximity of impacted canine to the adjacent teeth, have been identified as responsible for root resorption of the involved teeth. Ericson and Kurol^14^ (1988) concluded that the size of the follicle or the positioning of the lateral incisor showed no correlation with root resorption. However, it seems that unerupted canines increase the risk of root resorption in the adjacent teeth especially because of the physical proximity (<1 mm) between them^32^.

Another important sequelae related to orthodontic traction of impacted canines is the alveolar bone loss around the canine and the adjacent teeth as well as the final periodontal status^11,18,31^. The diagnosis of these complications and specially its extension can be critical in deciding the treatment plan to be adopted and the prognosis of the tooth impaction. In this regard, the advent of cone-beam computed tomography (CBCT) was extremely important, because it enabled minor changes to be detected with greater accuracy^24^. Thus, root resorption and alveolar bone loss of support tissues surrounding each tooth can now be more accurate and precisely diagnosed^24^. Ericson and Kurol^15^ (2000) demonstrated that the use of CBCT increased the detection of root resorption in approximately 50% compared with conventional x-ray exams.

Therefore, the aim of this study was to evaluate the long-term effects of orthodontic traction on root length and alveolar bone insertion in impacted canines and adjacent teeth.

## Material and methods

This retrospective study was approved by the Research Ethics Committee of Universidade do Sagrado Coração, under protocol number 541-211.

To perform sample size calculation, the root length measurement in the upper canines, lateral incisors and first premolars was conducted in a pilot study with six subjects. It was determined that the largest standard deviation of the difference between the tooth and its contralateral occurred in the buccal root measurement of the first premolar (1.50 mm). Thus, adopting an α of 5% and a power of 80% with a minimum mean difference to be detected of 10% in root length (1.47 mm), the sample size calculation showed that 10 subjects were necessary to achieve reliable results.

Initially, 28 subjects presenting with unilaterally impacted maxillary canine, palatal displaced, treated with the same surgical and orthodontic technique, were consecutively selected from an orthodontic graduate program and a private practice. The final total sample comprised 16 patients (nine males and seven females), mean initial age of 11 years and 8 months, who had CBCTs as final records because they were taken for third molars diagnosis purposes. At the end of the orthodontic treatment, all patients presented a mean age of 14 years and 2 months and were observed for a mean post-treatment period of 5 years and 11 months, varying from 1 to 12 years. As an inclusion criterion, the follow-up should be done at least 1 year after treatment.

Teeth from the impacted-canine side were assigned as Group I (GI), and contralateral teeth as control, Group II (GII). Patients were treated with the same traction protocol oriented by only one supervisor (LCF). The same professional performed the surgery to minimally expose the impacted canine crown. After that, a small perforation with a spherical Carbide bur (1/4) was done in order to pierce a 0.012” metallic wire ligature (Dentaurum GmbH & Co. KG, Ispringen, Baden-Württemberg, Germany), allowing the orthodontic traction. The advantages of the crown perforation are the lower risk of a second surgical procedure, since it is common when a bracket is bonded to a tooth under surgical conditions; the other advantage is related to the application of force in the long axis of the tooth that suffered traction in order to better control the direction of the traction procedure. In addition, less tissue manipulation and shorter surgery time are also observed^10^. After this procedure, the flap was repositioned and canine traction was performed with segmented arch mechanics by using 0.019x0.025” TMA wires exerting a continuous force deflection of 60 g. A stainless steel passive 0.032” transpalatal arch was used as anchorage and the impacted canines were orthodontically guided to its correct arch position.

In order to compare, in a long term-basis, root length and alveolar bone level in canines, lateral incisors and first premolars (both sides), measurements were performed in tomographic scans. The CBCT scans were taken in a post-treatment period of 5 years and 11 months (mean), with the following machines and acquisition settings: Prexion3D (PreXion Inc., San Mateo, Ca, USA), 90 kV, 4 mA, exposure time of 19 seconds, 8-cm-diameter field of view and 0.125-mm voxel size; i-CAT (Imaging Sciences International, Hatfield, PA, USA), 120 kV, 8 mA, exposure time of 40 seconds, 6-cm-diameter field of view and a 0.2-mm voxel size.

**Figure 1:**
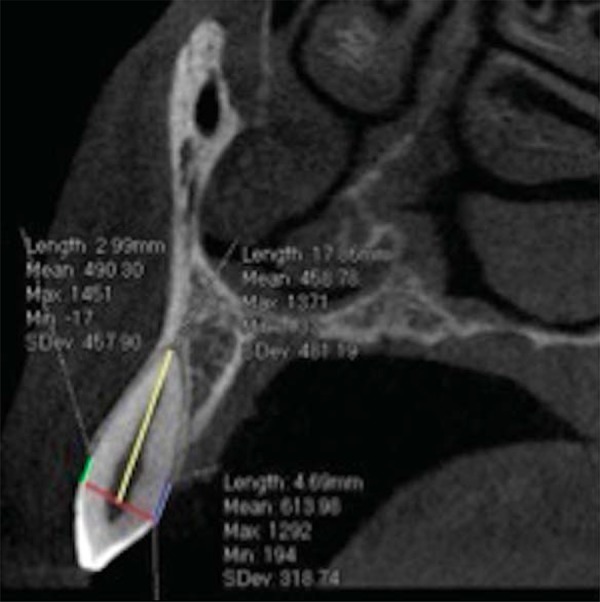
Sagittal view assessing root length (RL), buccal alveolar bone level (BABL) and palatal alveolar bone level (PABL) measurements. Yellow line (RL): from the cementoenamel junction (CEJ) to root apex; Green line (BABL): from the cementoenamel junction (CEJ) to buccal crest; Blue line (PABL): from the cementoenamel junction (CEJ) to palatal crest; Red line Line connecting the cementoenamel junction (CEJ)

**Figure 2:**
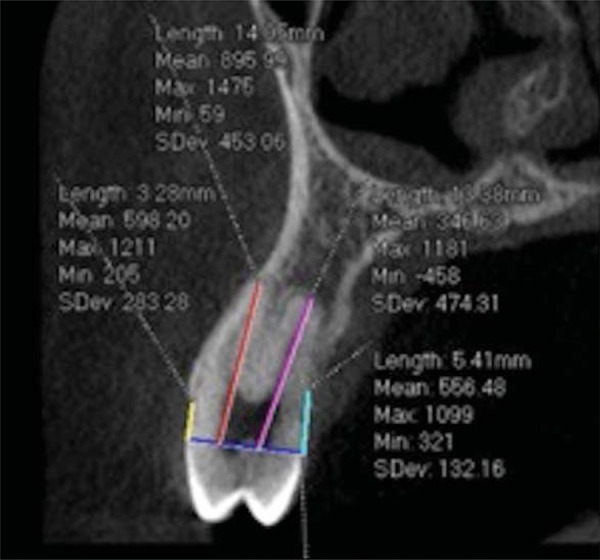
Coronal view assessing root length (RL), buccal alveolar bone level (BABL) and palatal alveolar bone level (PABL) measurements. Yellow line (BABL): from the cementoenamel junction (CEJ) to buccal crest; Red line (RL): from the cementoenamel junction (CEJ) to root apex; Pink line (RL): from the cementoenamel junction (CEJ) to root Apex; Blue line (PABL): from the cementoenamel junction (CEJ) to palatal crest; Purple line Line connecting the cementoenamel junction (CEJ)

The acquired images were converted into DICOM format (Digital Imaging and Communication in Medicine) and measurements were made using the Prexion 3D Viewer software (PreXion Inc., San Mateo, Ca, USA). The reconstructed images were analyzed with a 2-mm thickness parameter. In this study, the measurement method proposed by Kim, Park and Kook^21^(2009) was adapted for Prexion 3D Viewer software. To determine root length (RL), the distance from the cementoenamel junction (CEJ) to the apex was performed. In order to determine the buccal alveolar bone levels (BABL) and palatal alveolar bone levels (PABL), distances from CEJ to buccal and palatal alveolar crest were measured, respectively. Canines and lateral incisors measurements were performed on sagittal sections. Coronal sections were used to first premolars measurements (Figures 1 and 2).

### Statistical analysis

Intra-examiner systematic errors were assessed by applying paired t-test and random errors were analyzed with Dahlberg’s formula. To verify the normal distribution of the variables, Kolmogorov-Smirnov test was used. Data presented a normal distribution. The comparison of root length and alveolar bone level changes between groups was assessed by applying paired t-test, at a significance level of 5% (p<0.05). All statistical analyses were performed with Statistica software (Statistica for Windows 5.0; Statsoft, Tulsa, OK, USA).

## Results

### Root length

Regarding root length, results showed no statistically significant difference between groups (Table 1). The highest standard deviation of the difference between the means in the two groups was found in canine measurements (0.95 mm), and the lowest in the palatal root of the first premolars (0.40 mm). When comparing the two groups, GI showed decreased root length in 67.18% (0.63 mm) of the sample, with the largest reduction equivalent to 26% of the root length of the contralateral tooth.

**Table 1:** Intergroup comparison regarding root length measurements (paired t-test)

Teeth	G I		G II		dif.	P
	**mean**	**s.d**	**mean**	**s.d**		
LI(mm)	13.01	1.58	13.57	2.16	0.56	0.095ns
C(mm)	15.83	2.13	16.78	2.81	0.95	0.105ns
PM - B(mm)	13.77	1.62	14.41	1.77	0.64	0.083ns
PM - P(mm)	13.65	1.57	14.05	1.85	0.40	0.322ns

ns: non significant. C: canine; LI: lateral incisor; PM-B: buccal premolar; PM-P: palatal premolar.

### Alveolar bone level

Statistical analysis of the results showed no significant difference between groups. The highest standard deviation of the difference between the means regarding the buccal alveolar bone level (BABL) in the two groups was 0.03 mm found in the premolars (Table 2) and regarding the palatal alveolar bone level (PABL) was 0.39 mm, found in lateral incisors (Table 3). When comparing GI and GII, 56% of GI showed a decrease in BABL measurement and 58.3% in the PABL measurement. In three premolars, one in GI (impacted) and two in GII (control group), the absence of buccal alveolar bone was noted.

**Table 2:** Intergroup comparison regarding buccal bone level measurements (paired t-test)

Teeth	G I		G II		dif.	P
	**mean**	**s.d**	**mean**	**s.d**		
LI(mm)	9.38	2.97	10.30	2.21	0.92	0.166ns
C(mm)	12.49	3.15	12.13	4.50	-0.36	0.747ns
PM(mm)	9.03	3.95	9.06	4.11	0.03	0.981ns

ns: non significant. C: canine; LI: lateral incisor; PM: premolar.

**Table 3:** Intergroup comparison regarding palatal bone level measurements (paired t-test)

Teeth	G I		G II		dif.	P
	**mean**	**s.d**	**mean**	**s.d**		
LI(mm)	11.15	1.29	11.54	1.99	0.39	0.280ns
C(mm)	13.43	2.43	14.26	3.13	0.83	0.163ns
PM(mm)	10.86	2.02	10.14	2.77	-0.72	0.135ns

ns: non significant. C: canine; LI: lateral incisor; PM: premolar.

## Discussion

The need of significant tooth movement and the lengthy orthodontic treatment associated with forced eruption of ectopic canines may increase the susceptibility to root resorption and alveolar bone level changes in such patients.

Considering the sort of sequelae involved in orthodontic traction of impacted canines, the decision of using CBCT images in this study was based upon the higher accuracy and precision^24^of this method when compared with 2-D images. Furthermore, intraoral radiographs have disadvantages such as the difficulty of standardization and the image distortion^19,26^. Moreover, it would not be possible to visualize buccal and palatal alveolar bone levels when periapical films are taken, due to the superimposing of images, and, in lateral cephalograms, due to the teeth positions in the dental arch. In addition, such images do not allow the detection of fenestrations.

To perform root length measurement, either an ordinal scale (0-4) could be employed or the direct root measurement. Regarding the periodontal conditions, either the probing pocket depth^11^ could be performed or the measuring of the alveolar crest using periapical radiographs^19^. The method chosen in this study, to obtain the root length and alveolar palatal bone and level measurements, has been previously described by Kim, Park and Kook^21^ (2009), modified by Handelman^17^ (1996) and Beckmann, et al.^4^ (1998), which used the cement-enamel junction as reference. The accuracy of this method has already been proven earlier and the results of systematic error assessments, evaluated by paired t-test, showed no statistically significant difference, with exception of the buccal bone level of the lateral incisors, and the random error, assessed by applying Dahlberg’s formula was from 0.02 of the buccal alveolar bone level of the canines to 0.21 of the root length from lateral incisors.

Some important advantages of the closed-eruption technique associated with a canine crown perforation performed in this study are highlighted: reduced risk of a new surgical procedure; less tissue manipulation, especially the dental follicle (important framework for tooth eruption) and a mechanical advantage of allowing the application of force in the long axis of the teeth. A segmented arch and the use of transpalatal arch as an anchorage device was the selected mechanics for the orthodontic traction of impacted canines, as proposed by Lindauer and Issacson^23^ (1995). The canines were guided to erupt on the palate avoiding the contact with the roots of adjacent teeth and thus reducing the risk of root resorption.

Despite the results of this study had pointed out to a minimal decrease in the root length measurements of canines and adjacent teeth from GI compared with GII, no statistical significant difference was found. This slight difference, besides not statistically significant, has no clinical significance, since the maximum value of the comparison between sides was 0.95 mm. These results could be influenced by light forces application during the traction mechanics.

These findings reinforce the results found by Brusveen, et al.^8^ (2012) and Lempesi, et al.^22^ (2014) that there is no statistically significant difference among the groups. These authors, however, evaluated only the root length of the incisors. Woloshyn, et al.^31^ (1994) and Schmidt and Kokich^28^ (2007) evaluated through periapical radiographs the root length of the incisors, canines and first premolars. The only difference between these two studies was the surgical approach; in the first study they adopted the closed-eruption technique and orthodontic traction, similarly to our study, and in the second one, an apically positioned flap without orthodontic traction. Corroborating our results, the authors found a small decrease in root length, but without statistical significance. However, the root length of premolars, in the study of Schmidt and Kokich^28^ (2007), presented similar results among groups. A limitation in these studies was the use of periapical radiographs, which could influence the results because of the overlapping of buccal and palatal roots. Another difference between ours and Schmidt and Kokich’s study^28^ (2007) was the apically positioned flap technique employed on that research. We understand that not all canines can be treated by apical positioning of the flap without orthodontic traction, particularly those most ectopic positioned, in which orthodontic traction is a challenge for the orthodontist and may also influence on the root resorption induced by orthodontic treatment^22^.

Regarding the buccal and palatal alveolar bone level, although our results showed that comparing GI and GII, 56% of GI showed a decrease in BABL, this difference was not considered statistically significant. These results are similar to those reported by Schmidt and Kokich^28^ (2007) when evaluating the mesial and distal bone level using periapical radiographs. The same results regarding periodontal final condition (probing depth) were found by Caprioglio, Vanni and Bolamperti^11^ (2013) evaluating palatally impacted canines that suffered traction. On the other hand, Becker and Chaushu^3^ (2005) and Evren, et al.^16^ (2014), assessing the mesial and distal bone level in canines that suffered traction, also in periapical radiographs, found a statistically significant bone loss among groups. On the latest, not only palatally, but buccally displaced canines presented reduced bone levels compared with their contralaterals. It should be highlighted that palatally displaced canines, when suffering traction, may not compromise the periodontal status as those buccally displaced.

Some conflicting findings could be accounted by the method of evaluation, either CBCT scans or periapical radiograph, which could stand for such difference. Also, the traction protocol and the initial position of the canines could influence these results, since the role of adequate oral hygiene during appliance therapy may be significant^6,16,18^. More reliable results should be achieved in performing measurements in CBCT scans, but in two different periods, before and after treatment. The limitations of this study are that only the final CBCT images were available and its retrospective design. But exposing patients to unnecessary radiation should also be avoided, even considering only the maxillary area. Other limitation of our study is that the sample size seemed small (16 subjects), but the sample size calculation showed that 10 subjects were necessary to achieve reliable results.

It is important to emphasize that an early diagnosis is always better to prevent irreversible damages to the involved and adjacent teeth^9,12^. Even after an early diagnosis, in some cases it is necessary to perform tooth traction. Besides that, according to this research we can state that the traction protocol associated with the orthodontic corrective treatment did not negatively affect the periodontal status and the root length of the impacted canines and adjacent teeth.

## Conclusion

The treatment of impacted canines had minimal effect on root length and buccal and palatal alveolar bone levels, not only in orthodontic canines that suffered traction, but also in adjacent teeth (lateral incisor and first premolar), demonstrating a good long-term prognosis of this treatment protocol.
